# Design and evaluation of a mobile-based nutrition education application for infertile women in Iran

**DOI:** 10.1186/s12911-022-01793-x

**Published:** 2022-03-04

**Authors:** Mostafa Langarizadeh, Seyed Ali Fatemi Aghda, Azadeh Nadjarzadeh

**Affiliations:** 1 Department of Health Information Management, School of Health Management and Information Sciences, Iran University of Medical Sciences, Tehran, Iran; 2 Department of Health Information Management, School of Health Management and Information Sciences, Iran University of Medical Sciences, Tehran, Iran; 3 Nutrition and Food Security Research Center, Shahid Sadoughi University of Medical Sciences, Yazd, Iran; 4 Department of Nutrition, School of Public Health, Shahid Sadoughi University of Medical Sciences, Yazd, Iran

**Keywords:** Infertility, Nutrition education, Mobile health, Usability, Mobile app

## Abstract

**Background:**

The prevalence of infertility in Iran is higher than the world average. Furthermorte, education and nutrition are among the effective factors in improving the quality of life of women with infertility. According to the significant role of smartphones in people's lives as well as health education, the present study aimed to design and evaluate a mobile-based nutrition education application for infertile women.

**Methods:**

This quantitative research was conducted in two stages. Initially, the educational contents were determined based on a review of the literature. Later, the obtained contents were given to 10 nutritionists and five infertility specialists to determine the necessity of each item. In the next stage, the application prototype was designed based on the results of the first stage and distributed among 220 infertile women. After two months, the Questionnaire of User Interface Satisfaction was administrated to assess the usability of the developed application. The results were analyzed via SPSS software version 20.

**Results:**

According to the nutritionists and infertility specialists, the contents determined for the nutrition educational application were categorized under three general sections of user's demographic data, educational contents, required capabilities. The users' mean score of the application usability was calculated as 7.44 out of 9 indicating a good level of satisfaction.

**Conclusions:**

Nutrition education of women with infertility problems can play a significant role in improving their awareness and treatment outcomes. Due to the increasing use of smartphones, designing a mobile-based nutrition educational application can be of great benefit for women with infertility according to the cultural conditions and characteristics of each community.

## Background

The increasing prevalence of infertility, as one of the major health problems, has caused many challenges for individuals and communities. Although the global average rate of infertility is 5–7%, the reported rates of infertility in Iran vary from 13 to 20%. Such a wide range of reported data can be due to different definitions of infertility, inequality of the statistical population, and lack of accurate sampling. Infertility has various effects on economic, social, personal and psychological consequences [1, 2]. In this regard, infertility is known as one of the most difficult and critical stages of life affecting the structure of family and society [3, 4]. Recent studies showed that the prevalence of infertility varies under the influence of factors related to culture, health, geography, and human conditions, including the development of puberty, menstrual characteristics, history of contraception, previous pregnancies and their consequences, previous surgeries, especially pelvic surgery, history of infection, medication, and lifestyle, such as nutrition diet, weight, exercise [5–8].

Nutrition consists of taking nutrients that the body needs (protein, carbohydrates, fats, etc.), while avoiding poor eating habits (eating fast foods, soft drinks, etc.). Nutrition has been investigated broadly among women so that weight gain during pregnancy has a significant impact on the results of pregnancy [9, 10].

In this regard, Mostajeran et al. investigated the effects of using herbal medicines in traditional Iranian medicine as well as the lifestyle changes and modification of eating habitsin the current era [8]. Similarly, Dehghan et al. [11] elaborated on the effectiveness of taking herbal medicines as a complementary or even alternative treatment in infertile couples.

Recently, smartphone utilization has increased exponentially in various fields, such as agriculture, military, education, and health. Meanwhile, using mobile health (mHealth) and educational applications play a major role in developing accessibility to health services, reducing medical costs, and covering more target groups in different geographical areas [12]. Based on the literature, using mobile-based educational applications is more effective than traditional methods of education, such as lecturing and counseling. So, traditional administration of training courses along with using applications has been highly recommended [13, 14]. The term 'mHealth' refers to the use of smartphones or digital devices for training, managing, monitoring, diagnosing, and treating diseases [15–17].

In a prospective cohort study on 140 women with primary infertility, the findings showed that the participants' nutritional status affected infertility treatment outcomes so that nutritional interventions before attempting to treat infertility could increase the quality and quantity of ovaand sperms [18]. Recent studies [19–21] in the field of infertility also reported the significant effect of nutrition on the treatment of infertility indicating the educational need in terms of improving a healthy nutritional diet and lifestyle.

Few studies were conducted on the educational needs of women with infertility, especially in the field of nutritional problems in Iran. Furthermore, no nutrition education program has ever been developed for infertile women in this country. To this purpose, this study specifically addressed nutrition education in infertile women. The purpose was to collect appropriate educational contents in accordance with the culture and food preferences of Iranians according to the opinions provided by experts and specialists. The usability of the application was also assessed in order to consider the users' opinions.

## Methods

This study was conducted quantitatively in two phases. In the first phase, a researcher-made questionnaire was developed and administered among the experts to obtain the appropriate educational contents for designing the application. In the second phase, the application was designed and evaluated in terms of its usability based on the users' viewpoints. The research was carried out in Research and Clinical Center for Infertility, Yazd Shahid Sadoughi University of Medical Sciences, which is one of the most equipped and experienced centers of infertility in Iran. Given that Yazd province is located in the center of Iran, it receives a large diversity of patients with different cultures, customs, and traditions from all over the country.

### Phase A: Developing the educational contents

In the first stage of the study, the required educational contents and important nutritional details were obtained from library studies and published articles [22–25].

Initially, the published papers from January 2014 to November 2019 on springer, science direct, PubMed, and ISC databases were searched using the following keywords: infertility, nutrition education, mobile health. Later, the papers were analyzed and the initial draft of the questionnaire was developed.

A researcher-made questionnaire was designed to corroborate essential educational contents of the application based on the opinions provided by nutritionists and infertility specialists. This questionnaire contained three sections of demographic information, educational content, and required capabilities to work with the application. The respondents were supposed to answer the questionnaire based on a two-point Lickert Scale: Essential (1 score) and Non-essential (no score). Content validity of the questionnaire was confirmed by a panel of experts containing nine specialists (two PhD in medical informatics, two PhD in health information management, three nutritionists, and two infertility specialties). Reliability of the questionnaire was also corroborated using (13 nutritionists and 3 infertility specialties that)the KR-20 coefficient (KR = 0.89).

The questionnaire was distributed among 20 specialists. The inclusion criteria were working in Yazd Reproductive Sciences Institute and Avicenna Research Institute (ARI), having Iranian citizenship, and having work experience in the field of infertility. Data analysis was performed based on the frequency of items so that if an item was determined as 'essential' by at least 60% of the respondents (According to the previous studies [26, 27]), it was included in the application; otherwise, the item was removed. In order to analyze the data, SPSS software version 20 was run.

### Phase B: Designing and evaluating the application

The software was designed in Persian based on the approved educational contents from the first phase of the research. The application was developed based on visual basic (Vb) programming language, the Java Class Library (JCL), and Basic4 Android (B4A). Followed by performing the initial pilot test and fixing the bugs, the application was installed on smartphones of women with infertility (n = 220) who referred to the Research and Clinical Center for Infertility of Yazd from June to August 2021. The sample size was determined based on the number of available patients and their willingness to cooperate in the study. Based on the inclusion criteria, 24–41-year-old women with infertility problems who had been on no special diet and treated for at least 3 months were included in the study. After two months of using the application, the participants were asked to evaluate its usability via the standard Quiz questionnaire version 7. This questionnaire consists of two general sections: The first section includes demographic information of the respondents and the second section consists of six sub-sections: overall reactions to the software (6 Questions), screen (4 Questions), terminology and system information (6 Questions), learning (6 Questions), system capabilities (5 Questions), usability and user interface (2 Questions). The questionnaire was designed based on a 9-point Likert scale ranging from zero (the lowest) to nine (the highest).

## Results

In the first stage of the study, five questionnaires were missed out due to various reasons, such as the prevalence of COVID-19, specialists' lack of cooperation due to lack of time, and incomplete answers. As a result, 15 questionnaires were completed by the infertility specialists and nutritionists (response rate = 75%). The demographic information of the participants included their age, gender, specialization, and work experience (Table 1).

**Table 1:** Frequency distribution of the nutritionists and specialists' demographic information

Demographic information	Participants					
	Nutritionist		Infertility specialist		Total	
	n	%	n	%	n	%
*Age*						
40 <	2	13.3	0	0	2	13.3
40–50	5	33.3	3	20	8	53.3
50 >	3	20	2	13.3	5	33.3
*Gender*						
Female	6	40	3	20	9	60
Male	4	26.6	2	13.3	6	40
*Work experience*						
10 <	3	20	1	6.6	4	26.6
10–20	4	26.6	1	6.6	5	33.3
> 20	3	20	3	20	6	40

Contents of the designed application included nutrition educational information for women with infertility who were under treatment. According to the findings of this stage, the required educational contents were demographic information, educational contents, and required capabilities to work with the application (Tables 2, 3, 4).

**Table 2:** Frequency distribution of specialists about demographic information of the application users

Demographic information	Responses			
	Essential		Non-essential	
	n	%	n	%
Age	14	93.3	1	6.7
Height	12	80	3	20
Weight	13	86.6	2	13.4
Economic status	1	6.7	14	93.3
Educational level	10	66.6	5	33.4
Field of study	4	26.7	11	73.3
Residence	11	73.3	4	26.7
History of obesity	9	60	6	40

**Table 3:** Frequency distribution of specialists’ responses about the required educational contents of the application

Row	Educational content	Responses			
		Necessary		Unnecessary	
		n	%	n	%
1	*Definitions*				
	Infertility	15	100	0	0
	Dietary pattern	13	86.6	2	13.4
	Life style	12	80	3	20
2	*Disease and treatment instructions*				
	Hormonal diseases	11	73.3	4	26.7
	Chronic diseases	15	100	0	0
	Consumption of herbal tea and drinks	13	86.6	2	13.4
	Taking medication	15	100	0	0
3	*Diet and nutrition*				
	Importance of nutrition	12	80	3	20
	Effects of obesity	14	93.3	1	6.7
	Special treatment regimen	9	60	6	40
	The amount of calories consumed per meal	6	40	9	60
	Taking supplements and vitamins	10	66.6	5	33.4
	Allergy to certain foods	12	80	3	20
4	*Dietary habits*				
	Consuming fast foods	9	60	6	40
	Consuming fried foods	9	60	6	40
	Consuming fruits and vegetables	12	80	3	20
	Consuming simple carbohydrates	13	86.6	2	13.4
	Consuming of fats	11	73.3	4	26.7
	The interval between meals	7	46.6	8	53.4
	Number of meals per day	10	66.6	5	33.4
	Drinking carbonated beverages	12	80	3	20
	Drinking alcohol	8	53.3	7	46.7
5	*Personal activities and habits*				
	Having physical activity	10	66.6	5	33.4
	Tobacco use	13	86.6	2	13.4
	Using mobile phones	12	80	3	20
6	*Menstruation status*				
	Menstrual status	15	100	0	0
	Menstrual duration	15	100	0	0
	Menstrual severity	15	100	0	0

**Table 4:** Frequency distribution of specialists’ responses about the capabilities required to use the application

Capabilities	Responses			
	Necessary		Unnecessary	
	n	%	n	%
Calculate BMI	12	80	3	20
Resize content	9	60	6	40
Change the color of the content	4	26.6	11	73.4
Customizability For the user	7	46.6	8	53.4
Quick access menus	10	66.6	5	33.4

The specialists participating in the study were also asked to provide any additional suggestions about the application. Of 15 specialists, only 10 submitted suggestions, which were included in the application after some revisions (Table 5).

**Table 5:** Frequency distribution of specialists’ suggestions

Suggestions	Responses			
	Essential		Non-essential	
	n	%	n	%
Occupation	6	60	4	40
Characteristics of common foods	6	60	4	40
Introducing different types of diets	7	70	3	30
A common gynecological disease and the effects of nutrition on it	9	90	1	10
Introducing infertility clinics and centers in Iran	4	40	6	60
User's suggestions and comments	7	70	3	30

According to Table 5, contents related to introducing infertility clinics and centers in Iran were excluded since they did not receive the required acceptability score of 60% by specialists.

**Fig. 1:**
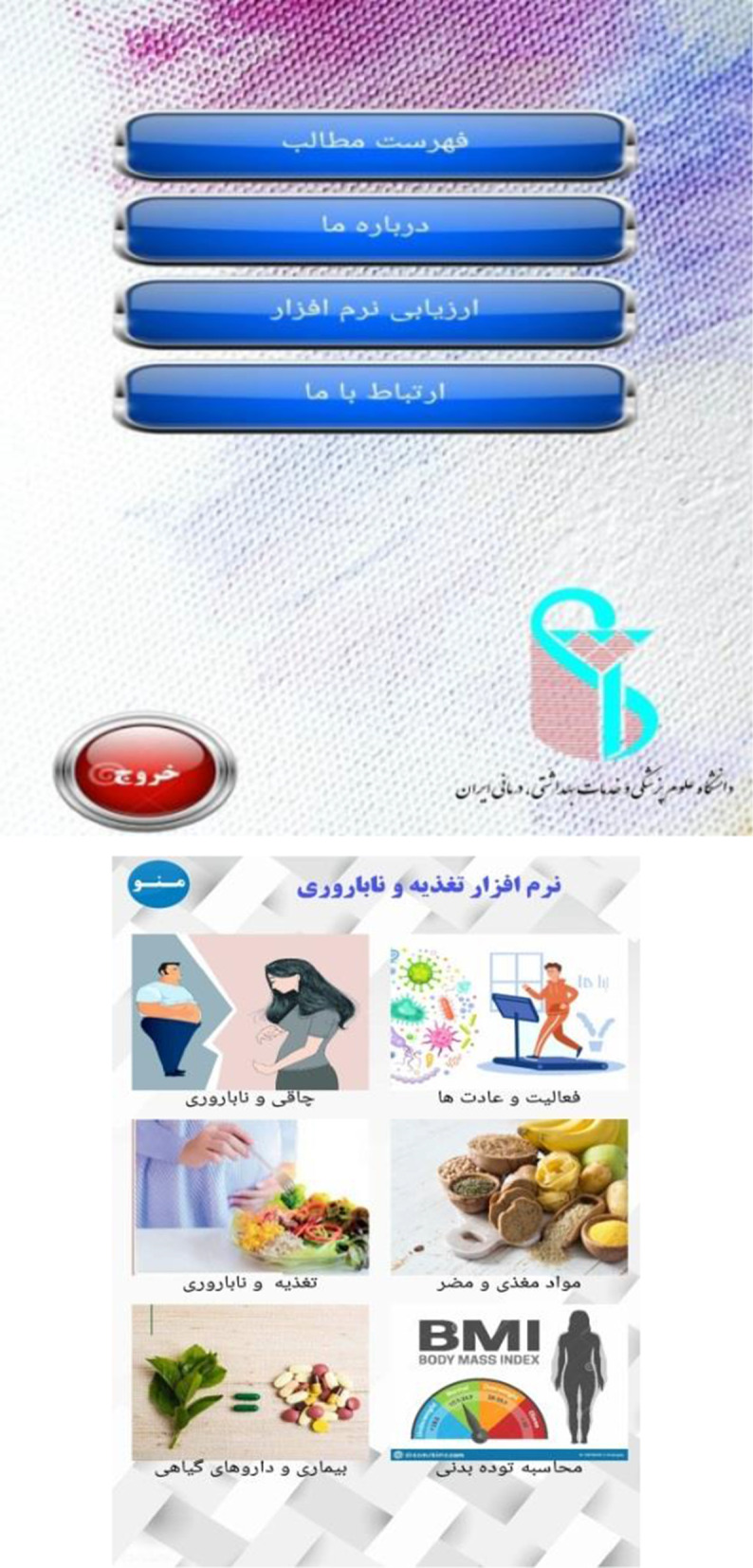
Several views of application pages

In the second stage of the study, the prototype of the mobile-based nutrition educational application for infertile couples was designed based on the results of the first phase. Figure 1 shows some parts of the application.

After implementing the prototype, 220 women with infertility were asked to use and evaluate the application prototype as end-users. The participants' demographic information included age, level of education, and place of residence (Table 6). Users' comments on different sections of the application are presented in Table 7.

**Table 6:** Frequency distribution of women's demographic characteristics

Variable	n	%
*Age*		
24–29	34	15.4
30–35	71	32.3
36–41	115	52.3
*Address (Residence)*		
Urban	137	62.2
Rural	83	37.8
*Education level*		
High school	17	7.7
Diploma	46	21
Associate degree	68	31
Masters and above	89	40.3

**Table 7:** Mean scoreof participants' answers to the questionnaire

Scale	Mean	SD
Overall reactions to the software	7.64	1.07
Screen	7.15	0.70
Terminology and system information	7.58	1.02
Learning	7.95	0.79
System capabilities	7.34	0.91
Usability and user interface	7.02	0.63

## Discussion

Given the extensive use of mobile health technology and the lack of adequate nutrition education programs for infertile women, nutrition education content was developed. Later, an application was designed based on the educational contents developed in the first phase and its usability was evaluated from the viewpoint of users. The majority of studies in the field of nutrition education investigated obesity, overweight, lifestyle, and its changes [18, 28–30].

Khalajabadi [31] investigated the required level of information and educational needs of women who were about to get married. According to the findings, women were at a low level of knowledge and awareness about fertility, which justifies the need for education among women of marriageable age.

The information reported by Greiger [32] on consuming fruits, fast foods, and beverages was used in developing the present application. Our study was almost identical to the research conducted by Pospteningram et al. [33] in terms of data collection and information acquisition procedures. The difference was that our initial data were corroborated by some professionals and specialists but their data were piloted on a sample of community members. Furthermore, we aimed to provide infertile women with nutritional information via a mobile application.

In the present study, the data collection procedure included administration of a researcher-made questionnaire among experts and physicians to confirm necessity of the items determined through library studies and maximum literature review while Silva et al. [34] only investigated infertile couples.

Lemoni et al. [35] examined the information needs of individuals seeking reproductive services in Canada by designing an application. Their purpose was to investigate information search cases about reproductive services and infertility based on the individual's needs. They also investigated the relationship between meeting information needs and psychological outcomes. In contrary to this study, we paid special attention to the nutritional needs of the application users, our application was designed according to the Iranian lifestyle and culture, and the developed application was evaluated using the standard Quiz questionnaire. In our study, the importance of accurate, relevant, and up-to-date information as well as establishment of appropriate relationship among people involved in medicine, software engineering, and medical informatics were observed, which were less considered by other studies.

Although Zwingerman et al. [36] developed and evaluated a mobile application in the field of infertility management. In this study, we tried to meet inadequacies of the application developed by these researchers (such as incorrect information and software deficiencies) using viewpoints of professionals in the fields of infertility, nutrition, and software engineering.

Ford et al. [37] examined the relationship between fertility and infertility knowledge among Australian women over 18 years of age using a mobile-based education program.

In this study, to evaluate the software usability and determine the users' level of satisfaction, the standard Quiz questionnaire version 7 was administered. Considering the prevalence of COVID-19 and lack of cooperation by many patients, the questionnaire was distributed using electronic and hard copies. As indicated in Table 7, all mean scores rated by users were higher than six in all sections maintaining the users' good level of satisfaction about application usability.

Ghazi Saeedi et al. [38] designed a self-care application for patients with heart failure using the Java programming language and the IntelliJ IDEA. Our application was designed via B4A, Vb programming language, and Jcl.

Similarly, Valente et al. [39] designed and evaluated a glaucoma application to facilitate the treatment process based on Android and IOs operating systems. However, in our study the application was designed for the Android operating systems.

Lemoni et al. [35] analyzed the information needs of individuals regarding fertility and infertility services using an Infotility application. This study varied from our research considering the type of application software (Infotility), evaluation questionnaire user mobile application rating scale (UMARS), number of samples, duration of application use, and analysis method.

Mendrachia et al. [40] investigated the role of mobile-based nutrition education application software in promoting and monitoring vegetable consumption in patients. In this systematic review study, researchers evaluated an application software using a mixed-method design by three independent evaluators. However, software evaluation was performed by real users and the Quiz questionnaire in our study.

Various dimensions of the Smarter Pregnancy Coaching Software were examined in two separate studies by Sting et al. In the first investigation, compliance and effectiveness of a nutrition application designed based on mHealth software and the role of accurate education in fertility of couples undergoing in vitro fertilization were studied [41]. The second study evaluated effectiveness of the developed application in reducing the cost of infertility treatment and its cost-effectiveness [42]. Both of these interventional studies aimed to evaluate the impact of educational contents on improving the users' level of information and reducing the treatment costs. According to their objectives, Sting et al. did not specify their evaluation methods; they only referred to the evaluation of nutrition contents and user behaviors by the questionnaire. Our research varies in terms of the administered questionnaire, duration of using application, and number of participants.

Some limitations of the present study were the non-generalizability of the current results to the entire Iranian infertile community, the lack of information needs assessment of the end-users (Women with infertility) to determine the required educational content application they need.

lack of cooperation by some specialists in completing the questionnaire due to lack of time or awareness about the study domain. This problem was largely resolved by providing them with a clear explanation about the study objectives and procedures. Due to COVID-19 pandemic, many patients delayed their treatment or used remote treatment, which hampered evaluation of the developed application. This limitation was eliminated by designing an electronic format of the questionnaire. The language of this program was Persian, which was selected according to the users' culture and research area. Further research is recommended on the development of a nutrition education software for infertile and other diseases.

## Conclusion

The aim of this study was to determine the required content for designing a nutrition education application. Later, usability of the application was assessed in terms of the viewpoints provided by women with infertility problems. The application was designed for Android operating systems. The results of the application usability assessment indicated appropriateness of the application. Consequently, this application can be considered as a tool for designing and creating broader programs in the field of treatment. Furthermore, institutions and organizations providing health care services can use this application to enhance their patients' information, help them to improve their lifestyle, and promote their quality of life.

## Data Availability

The data used and analysed during the current study are not publicly available due Iran University of Medical Sciences policy but are available from the corresponding author on reasonable request.

## Abbreviations

QUIS: Questionnaire of user interface satisfaction
B4A: Basic4Android
